# Personality Measures Link Slower Binocular Rivalry Switch Rates to Higher Levels of Self-Discipline

**DOI:** 10.3389/fpsyg.2016.02008

**Published:** 2017-01-05

**Authors:** Anna Antinori, Luke D. Smillie, Olivia L. Carter

**Affiliations:** Melbourne School of Psychological Science, University of MelbourneParkville, VIC, Australia

**Keywords:** visual awareness, binocular rivalry, personality, individual differences

## Abstract

In this paper we investigated the relation between personality and the rate of perceptual alternations during binocular rivalry. Studies have demonstrated that slower rivalry alternations are associated with a range of clinical conditions. It is less clear whether rivalry dynamics similarly co-vary with individual differences in psychological traits seen across non-clinical population. We assessed rivalry rates in a non-clinical population (*n* = 149) and found slower rivalry alternations were positively related *r*(149) = 0.20, *p* = 0.01 to industriousness, a trait characterized by a high level of self-discipline using the Big Five Aspect Scales (BFAS). Switch rates were also negatively related *r*(149) = −0.20, *p* = 0.01 to cognitive disorganization, a schizotypy trait capturing schizophrenia-like symptoms of disorganization using the Oxford-Liverpool Inventory of feelings and Experiences (O-LIFE). Furthermore, we showed that that these relations with personality were unaffected by the inclusion or exclusion of mixed percept in the response analysis. Together these results are relevant to theoretical models of rivalry investigating individual differences in rivalry temporal dynamics and they may reduce concerns about the impact of task compliance in clinical research using rivalry as a potential diagnostic tool.

## Introduction

Under normal viewing conditions each of our eyes accesses a slightly different image of the world. These different images, however, are typically fused into one seamless percept by our brain. A completely different perceptual experience can be induced when two incompatible images are presented to each eye simultaneously. Under these conditions observers typically experience a phenomenon known as binocular rivalry (Wheatstone, 1838). During binocular rivalry one's perceptual experience will alternate over time, despite the physical stimulus remaining constant, as the two visual representations *rival one* another for exclusive dominance (for review see Blake and Logothetis, 2002). These perceptual alternations have been described as a stochastic phenomenon, with individuals alternating on average every 1–2 s (Levelt, 1967). In other words, although alternations are not predictable, the switch occurs in a semi-regular fashion. This pattern of alternations between exclusive dominance (average switch duration) follows temporal dynamics that are highly stable within the same person, but vary between people (Aafjes et al., 1966; Miller et al., 2010). Rivalry is therefore a phenomenon with pronounced individual differences.

To date, the majority of rivalry research has focused on unraveling the general principles of rivalry by looking at the effect of visual stimulus features on rivalry temporal dynamics. For example, by investigating spatial frequency (O'shea et al., 1997), stimulus size (Kang, 2009), motion velocity (Knapen et al., 2007), and luminance (Wolfe, 1983). While such research is paramount to the advancement of the discipline, factors underling the striking degree of individual differences in rivalry alternation rate are equally important as they similarly impact the perceptual experience of the observer. The few studies that have explored individual differences in the dynamics of binocular rivalry identify a number of interesting associated factors (for a review see Kleinschmidt et al., 2012). For example, differences in rivalry rate have been linked to variations in density and thickness of the parietal cortex (Kanai et al., 2010), activity in early visual area (Yamashiro et al., 2014), gaze movements using static binocular rivalry gratings (Hancock et al., 2012; however, when using drifting binocular gratings the results was not replicated by Law et al. (2015) and GABA concentration in visual cortex (van Loon et al., 2013). Studies have also suggested a genetic basis for this variability, supported by identical twins showing greater similarity in rivalry dynamics than non-identical twins (Miller et al., 2010).

Research from clinical populations has demonstrated such clear differences in alternation rate when compared to healthy populations, that it has been suggested that binocular rivalry might have value as a diagnostic tool (Ngo et al., 2011). For example, converging evidence suggests that bipolar patients have significantly slower alternation rates than healthy control participants (Pettigrew and Miller, 1998; Nagamine et al., 2009; Ngo et al., 2011; Vierck et al., 2013). Deviance from normal rivalry rate has also been associated with schizophrenia (Frecska et al., 2003; but also see Wright et al., 2002; Miller et al., 2003), ADHD (Amador-Campos et al., 2013; Aznar Casanova et al., 2013), autism spectrum disorder (Robertson et al., 2013; but also see Said et al., 2013), and a trend toward slower rivalry between migraine events (Wilkinson et al., 2008). Despite results not always being replicated between laboratories, together these studies show a general pattern of slower perceptual alternations associated with a number of the clinical conditions. To date the only study to show clinically relevant increases in rivalry rate, identified a positive correlation with rivalry rate and anxiety measures (within a healthy population) with those scoring high in anxiety having the fastest switching rate (Nagamine et al., 2007).

While there is considerable consistency in the literature, a few studies have reported conflicting results. For example, Miller et al. (2003) observed no significant differences in rivalry rates between people with schizophrenia and healthy control, while Wright et al. (2002) reported slower rivalry alternations in people with schizophrenia and first-degree relatives of people with schizophrenia. In a similar vein Frecska et al. (2003) reported that people with schizophrenia maintain slower perceptual alternations also during a variation of the classic rivalry paradigm involving dichoptic stimulus alternation (DSA). This lack of replicability may reflect unavoidable practical limitations inherent in clinical research, such as misdiagnosis, comorbity with other disorders, confounding drug effects, and reduced patients' task compliance. In addition, this variability in findings and the lack of exclusivity in the relationship between rivalry temporal dynamics and specific clinical conditions suggests that clinical research may not be the best avenue to pursue for a better understanding of rivalry variability. Therefore, the current study aimed to explore the contribution of personality traits to rivalry temporal dynamics, outside the clinical domain.

The objective of this paper was to examine whether perceptual alternations correlate with non-clinical personality traits within a healthy sample. Mounting evidence suggests that clinical populations differ from the general population largely by degree rather than by kind (Haslam et al., 2012), and that many aspects of psychopathology can be organized together with individual differences in normal personality within a single structural framework (O'Connor and Dyce, 2001; Markon et al., 2005). Gaining a better understanding of correlations between personality and rivalry rate would therefore complement and extend the emerging line of research investigating rivalry as a diagnostic tool. In addition, demonstration of coherent patterns of variation in binocular rivalry has important implications for current biological models of binocular rivalry. This is because most common models of rivalry such as the classical reciprocal inhibition model (Blake, 1989) or hybrid model (Dayan, 1998; Freeman, 2005; Tong et al., 2006) are currently unable to account for associations between rivalry dynamics and complex human characteristics, such as personality.

To provide a broad exploration of the relation between personality traits and the average rate of switching in binocular rivalry, we have identified two relevant frameworks. First, an extensive body of literature has repeatedly demonstrated that most variations in personality can be organized in terms of five trait domains, often known as the “Big Five”: extraversion, openness, conscientiousness, agreeableness and neuroticism (or emotional stability; Digman, 1990; Costa and McCrae, 1992; Goldberg, 1993; John and Srivastava, 1999). These domains represent the major lines of covariation among all personality traits, and can be recovered through factor analyses of personality questionnaires that were not designed to measure the Big Five (Markon et al., 2005). Assessment of these broad trait domains, as well as narrower traits that lie at a lower-level of the structural trait hierarchy (DeYoung et al., 2007), allows us to cast a wide net in our exploration of the relationship between perceptual alternations and personality traits. Secondly, we aimed to unravel differences and similarities in perceptual alternations in healthy individuals and clinical populations. To this end, we focused on schizotypal traits due to its relevance to perceptual phenomenon. For example, positive schizotypy is often associated with apophenia—the detection of meaningful patterns in random visual stimuli (DeYoung et al., 2012). There is a longstanding and ongoing debate regarding the extent to which schizotypy scores reflects a continuum between normal variations in personality and diagnosable schizophrenia (Chapman et al., 1995; Claridge et al., 1996), and two opposing models can be identified in the literature. That is, the quasi-dimensional model, advocating that the presence of schizotypy leads to a higher risk of developing psychopathology (Rado, 1953; Meehl, 1962); and the fully dimensional model, advocating that schizotypy is a personality dimension, separated from pathology (Claridge, 1997). Regardless, one thing is clear: individuals high in schizotypy traits do share a number of perceptual and cognitive characteristics of schizophrenia patients, although manifested in a milder way (Cuesta et al., 2001).

With respect to binocular rivalry and schizophrenia, some studies showed that rivalry alternations deviate from the norm in this patient population (Sappenfield and Ripke, 1961; Fox, 1965; Wright et al., 2002; Frecska et al., 2003). Accordingly, we expected individuals scoring high in schizotypy traits to show an analogous, though less extreme pattern. A further independent question that we asked in this sample was the extent to which the *mixed* percept (i.e., when the two rivalry stimuli are perceived as fused into one scrambled or superimposed image) affects one's dominance durations. This question is of particular relevance given that several clinical studies reported an increase in mixed percept together with slower perceptual alternations (Wilkinson et al., 2008; Aznar Casanova et al., 2013; Robertson et al., 2013). For example, Robertson et al. (2013) found that in individuals with autistic spectrum condition rivalry is characterized by slower perceptual switching combined with an increased duration of mixed reported. Surprisingly, before this study no research has investigated whether a similar trend—increased mixed periods co-occurring with slower switching—exists in the general population. Furthermore, it is not clear to what extent mixed impact the relations that rivalry temporal dynamics have with other stable factors within the same person. This is important for two reasons. First, although mixed can be reduced by manipulating stimuli size (Blake et al., 1992) it is impossible to completely eliminate it. Thus, it is important to better understand whether it has a strong impact on the calculation of one's switching rate. To tease apart the influence of mixed in our findings, here we investigated whether the same relations hold when mix is excluded from mean percept duration and when mixed is included in mean percept duration.

## Methods

### Participants

We recruited 160 University of Melbourne undergraduate students, who participated in the research for course credit. All participants had normal or corrected to normal vision. We excluded 11 participants prior to analysis: 6 because they saw either a sustained mixture of the two stimuli or one of the stimuli dominated for more that 70% of the time; 5 because they responded for less than 50% of the total duration of the trial. There was therefore a final N of 149 participants (30%Male); age (*M* = 19.49, *SD* = 2.92).

### Personality trait measures

#### Big five personality traits

The Big Five Aspect Scales (BFAS) DeYoung et al. (2007) is a 100-item measure of the Five Factor Model (McCrae and Costa, 1987). Each of the trait *domains* is divided into two lower level *aspects* (DeYoung et al., 2007). Respondents indicate the extent to which they agree or disagree with each of the items on a 5-point rating scale ranging from 1 (strongly disagree) through 3 (neutral) to 5 (strongly agree). Cronbach's alpha for each trait domain and its component was acceptable (Table 2).

#### Schizotypal personality

The Oxford-Liverpool Inventory of feelings and Experiences (O-LIFE) is a 104-item developed by Mason et al. (1995) measuring 4 components of schizotypy: Unusual Experiences, Cognitive Disorganization, Introvertive Anhedonia, and Impulsive Nonconformity. Participants respond to each item with a two-choice format (YES/NO). Cronbach's alpha was acceptable for each O-LIFE scales (Table 2).

### Binocular rivalry

#### Apparatus and stimuli

The rival targets were stationary green and red gratings (each grating subtend a visual angle of 2°, with a spatial frequency of 4cpd) oriented ±45° from vertical, with a circular frame. Stimuli were generated in Matlab, using the Psychophysics Toolbox extension (Brainard, 1997; Pelli, 1997) and displayed on an Apple mac computer monitor (23-inch monitor, 60 Hz frame rate, 1280 × 800 pixel resolution), stimuli were viewed through a mirror stereoscope (viewing distance 33 cm).

#### Instructions

Participants were instructed to continuously report what they were experiencing via key press: while perceiving the red or green grating they had to press and hold down the “Left Arrow” key or the “Right Arrow” key respectively. Participants were instructed to report any instances of mixed percept (time where the two stimuli appeared as a grid or patchwork combination of the two percepts) by pressing the left and right arrow keys simultaneously.

#### Response recording

Data were recorded in a single 120 s trial with observer responses (state of the keyboard) sampled every 220 ms. Prior to the experimental task, participants underwent a 60 s training session to ensure they understood the instructions. Mean percept duration was calculated as the average duration of time (seconds) that participants reported uninterrupted dominance of either one of the rival targets. Mixed percept was recorded, but removed from analysis of the mean percept duration. However, to investigate the influence of mixed on mean duration, we also calculated mean percept duration with mixed percept. In this case, mean percept duration was calculated as the average duration of time between the participant's report of full dominance of one grating and their next report of full dominance of the alternative grating stimuli. To be considered as an instance of mixed response “Left arrow” key and “Right arrow” key had to be pressed simultaneously. To reduce the impact of minor finger adjustments or sluggish transitions between the left and right button press (resulting in a brief overlap of both buttons being pressed) we only included mixed responses that spanned more than 220 ms (2 consecutive keyboard response samples) in our mixed percept analysis.

## Results

### Binocular rivalry rate and personality

As the data for percept duration was significantly non-normal (Shapiro-Wilk test *p* < 0.001) a Spearman's rank-order correlation was used to examine the association between binocular rivalry mean percept duration (*M* = 2.13; *SD* = 0.72) and personality trait measures. Because multiple comparisons were used to test the relationship between personality traits and mean percept duration, it was necessary to control for type I errors. We used the procedure introduced by Benjamini and Hochberg (1995) that is similar to Bonferroni-type corrections, but is more appropriate for data of this type as it also reduces the possibility of type II errors by controlling for the false discovery rate (Nakagawa, 2004). Only two-tailed tests were used. With respect to Big Five personality traits, a signification positive correlation was found between *Industriousness* and percept duration *r*_*s*_(149) = 0.20, *p* = 0.01. With respect to schizotypal traits a significant negative correlation was found between *Cognitive Disorganization* and percept duration *r*_*s*_(149) = −0.20, *p* = 0.01. Other traits of either the Big Five or schizotypal did not correlate to percept duration (Table 1). It is worth noting that these seemingly-small associations are close to the average effect size within personality research for variables that do not share method variance (i.e., *r* = 0.21; Richard et al., 2003), which falls within the middle third of effect sizes in the whole of psychology (Hemphill, 2003). Because a conceptual similarity exists between cognitive disorganization and industriousness—they are traits capturing opposite qualities *r*(149) = −0.56, *p* < 0.001—we suspected that the relationships found between percept duration and these traits were driven by a similar underlying factor. Further analysis confirmed this. When controlling for industriousness on the relationship between cognitive disorganization and percept duration, the partial correlation was not significant *r*_*s*_(149) = −0.07, *p* = 0.45. Similarly when controlling for cognitive disorganization on the relationship between industriousness and percept duration, the partial correlation was not significant r_*s*_(149) = 0.09, *p* = 0.31.

**Table 1:** **Spearman Correlation Coefficient of mean percept duration and Personality Traits measured using the BFAS and O-LIFE scales**.

	**Scales**	**Mean percept duration**	
		***r***	***p***
**BFAS**	***Openness/intellect***	−0.08	0.34
	Openness	−12	0.14
	Intellect	−0.04	0.67
	***Conscientiousness***	0.16	0.05
	Orderliness	0.08	0.31
	Industriousness	0.20^*^	0.01
	***Extraversion***	−0.05	0.57
	Enthusiasm	0.03	0.67
	Assertiveness	−0.10	0.22
	***Agreeableness***	0.06	0.49
	Politeness	0.08	0.36
	Compassion	0.02	0.77
	***Neuroticism***	−0.06	0.49
	Withdrawal	−0.12	0.15
	Volatility	0.01	0.92
**O-LIFE**	***Unusual Experience***	−0.11	0.20
	***Cognitive Disorganization***	−0.20^*^	0.01
	***Introvertive Anhedonia***	−0.02	0.81
	***Impulsive Non Conformity***	−0.15	0.08

* *Indicate correlations remaining significant after Benjamini & Hochberg's procedure; false discovery rate [FDR] = 0.2; p < 0.05 (Two-tailed)*.

**Table 2:** **Means, Standard Deviations, and intercorrelations among all Personality Trait Measures (BFAS and O-LIFE)**.

**Variables**	**1**	**2**	**3**	**4**	**5**	**6**	**7**	**8**	**9**	**10**	**11**	**12**	**13**	**14**	**15**	**16**	**17**	**18**	**19**
1. **Openness/Intellect**	(0.86)																		
2. Openness	*0.84*	(0.79)																	
3. Intellect	*0.84*	*0.42*	(0.82)																
4. **Conscientiousness**	0.06	−0.05	*0.15*	(0.87)															
5. Orderliness	−0.12	−0.13	−0.07	*0.85*	(0.81)														
6. Industriousness	0.*22*	0.04	*0.33*	*0.86*	*0.85*	(0.87)													
7. **Extraversion**	0.*31*	*0.17*	*0.35*	*0.27*	0.46	*0.32*	(0.91)												
8. Enthusiasm	*0.17*	0.11	*0.18*	*0.21*	0.14	*0.20*	*0.85*	(0.86)											
9. Assertiveness	*0.36*	*0.19*	*0.42*	*0.25*	0.08	*0.34*	*0.88*	*0.49*	(0.89)										
10. **Agreeableness**	*0.23*	*0.18*	*0.19*	0.12	0.05	*0.16*	*0.17*	*0.31*	−0.01	(0.84)									
11. Politeness	−0.01	−0.02	0.01	*0.16*	0.08	*0.19*	−0.13	0.08	−*0.29*	*0.81*	(0.72)								
12. Compassion	*0.37*	*0.32*	*0.29*	0.05	0.01	0.08	*0.38*	*0.42*	*0.24*	*0.84*	*0.36*	(0.87)							
13. **Neuroticism**	−*0.26*	−0.08	−*0.35*	−*0.24*	0.05	−*0.46*	−*0.31*	−*0.25*	−*0.29*	−0.13	−*0.17*	−0.05	(0.90)						
14. Withdrawal	−*0.32*	−0.010	−*0.43*	−*0.24*	0.07	−*0.48*	−*0.44*	−*0.29*	−*0.47*	0.01	0.06	−0.04	*0.87*	(0.81)					
15. Volatility	−*0.14*	−0.04	−*0.19*	−*0.19*	0.02	−*0.34*	−0.12	−*0.16*	−0.06	−*0.22*	−*0.34*	−0.05	*0.89*	*0.54*	(0.88)				
16. **Unusual experience**	*0.**16***	***0.30***	−0.03	−0.08	−0.06	−0.07	−0.03	−0.11	0.05	−0.13	−0.14	−0.08	0.13	***0.18***	0.07	(0.87)			
17. **Cognitive disorganization**	−***0.27***	−0.03	−***0.44***	−***0.37***	−0.06	−***0.56***	−***0.45***	−***0.33***	−***0.45***	−0.08	0.00	−0.13	***0.70***	***0.73***	**0**.***50***	0.*32*	(0.90)		
18. **Introvertive anhedonia**	−***0.21***	−***0.21***	−***0.15***	−0.11	−0.07	−0.011	−***0.64***	−***0.68***	−***0.43***	−***0.27***	−0.06	−***0.36***	***0.18***	***0.22***	0.11	0.12	*0.38*	(0.81)	
19. **Impulsive non conformity**	0.06	0.013	−0.04	−***0.35***	−**0.25**	−***0.34***	***0.14***	0.04	***0.19***	−***0.33***	−***0.43***	−0.12	***0.32***	***0.15***	**0.40**	*0.37*	*0.35*	0.01	(0.70)
Mean	3.58	3.68	3.50	4.20	3.30	3.11	3.47	3.67	3.26	3.97	3.85	4.08	2.84	2.95	2.73	10.48	12.09	5.73	9.84
Standard deviation	0.54	0.64	0.65	0.61	0.70	0.70	0.57	0.63	0.69	0.44	0.51	0.58	0.63	0.69	0.75	6.02	6.07	4.13	4.13

*Cronbach's alphas for all variables are presented in parentheses on the diagonal of the correlation matrix, p < 0.05 for all correlations; correlations between scale of different personality measure are presented in **boldface**; correlation between traits of the same personality measure are presented in italics*.

### Binocular rivalry rate and mixed percept

**Figure 1:**
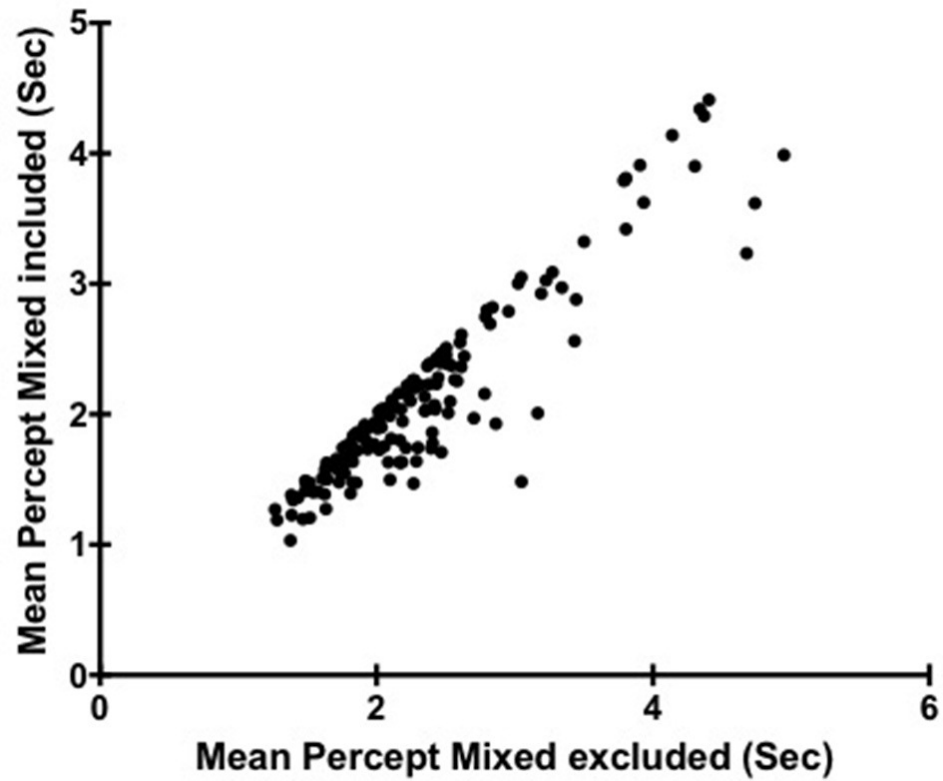
**Scatter plot showing the relationship between Mean Percept Duration when Mixed is included and Mean Percept when Mixed is excluded**. Each point corresponds to a different participant (*N* = 149).

To investigate the impact of including or excluding mixed percept affected mean percept duration in our analysis of individual differences of mean percept duration we first plotted mean durations from individual's calculated with and without mixed percept (Figure 1). To test whether there was any significant effect of these different analysis methods a Spearman's rank−order correlation was performed between percept durations when time reporting mixed was excluded (*M* = 2.13; *SD* = 0.72 as stated above), or included (*M* = 2.36, *SD* = 0.77) with results showing *r*_*s*_(149) = 0.89, *p* < 0.001. This indicates that overall in our subject group, individuals found to have a relatively faster (or slower) switch rate ended up being ranked in a similar order regardless of whether mixed percept was included.

**Figure 2:**
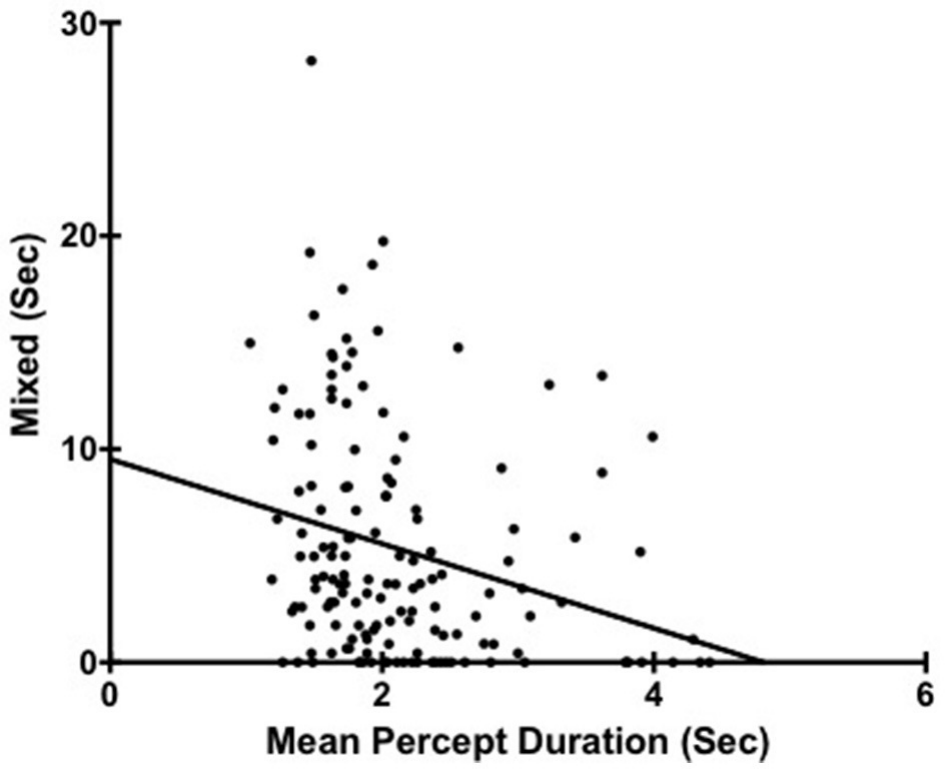
**Scatter plot showing the relationship between Mean Percept Duration and Mixed**. Each point corresponds to a different participant (*N* = 149).

We did find a significant correlation between percept duration (with mixed excluded form calculation) and total amount of mixed percept reported *r*_*s*_(149) = −0.34, *p* < 0.001 (Figure 2). That is, people reporting more mixed were characterized also by shorter exclusive percept duration. However, in terms of the main focus of this current study looking at personality and mean percept duration it is important that the original relationships were similarly seen between percept duration and both industriousness *r*_*s*_(149) = 0.18, *p* = 0.03 and cognitive disorganization *r*_*s*_(149) = −0.20, *p* = 0.02.

## Discussion

Using the Big Five model and schizotypy measures our findings indicate that slower rivalry switch rates are positively correlated with industriousness and negatively correlated with cognitive disorganization. The finding that rivalry rate was slower in individuals scoring high in conscientiousness was unexpected. Conscientiousness is one of the broad domains of the Big Five and is divided into two aspects: industriousness and orderliness, with the latter aspect driving the correlation with slower alternations. Examples of industriousness items are “I get things done quickly,” and items with reversed score, such as “I am easily distracted.” The conscientiousness trait aims to capture aspects related to one's determination and ability to control immediate impulses to achieve long-term rewards (DeYoung et al., 2007). Because many of the clinical populations previously found to have slow rivalry alternations are often considered to have difficulty maintaining task focus, it was surprising that slower rivalry rates were associated with greater industriousness in our healthy population. One important implication of this result, therefore, is that it argues against the concern that the slower rates of rivalry in these clinical populations is a spurious finding resulting from *missed* reports of perceptual transitions due to a lack of task compliance or sustain attention (i.e., lower industriousness) in these populations. Our results suggest that the opposite relationship exists, with individuals with the lowest scores on industriousness showing the fastest rates of rivalry.

Faster alternations in this study were also associated with higher scores in cognitive disorganization. This represents one of the main dimensions of schizotypy, characterized by schizophrenia-like symptoms of disorganization such as loose conceptual boundaries (Mason et al., 1995). Upon examining the associations among mean percept, cognitive disorganization and industriousness, it was clear that those associations really reflected a single effect. This is important as it means we have effectively replicated our main findings using two different measurement tools. Across inversely related traits faster switching was associated with increased cognitive disorganization and decreased industriousness. The link between cognitive disorganization and conscientiousness has been previously found in research investigating the relationship of schizotypy traits with broader trait dimensions. Indeed this is in line with the fully dimensional approach advocating schizotypy as a personality trait observable in a non-clinical population (Asai et al., 2011). In our data industriousness (a lower-level trait of conscientiousness) was significantly correlated with cognitive disorganization and when we controlled for either traits, the relation with mean percept no longer existed.

With respect to schizotypy our data provides the first evidence that a relationship exists between schizotypal traits and rivalry alternations within a normal population. This is in contrast to research exploring an association between schizophrenia (as opposed to schizotypy) and rivalry alternations, which has previously been reported as either showing slower (Wright et al., 2002; Frecska et al., 2003) or no change in rivalry rate (Miller et al., 2003) compared to healthy controls. Our findings add another level of complexity to this issue and suggest that in a non-clinical population, rivalry alternations may be influenced by different factors. In those studies investigating schizophrenia and rivalry alternations, however, the authors compare people with schizophrenia and control (non-clinical population). Because the cognitive disorganization scale was the only schizotypy trait correlating with rivalry temporal dynamics, future studies investigating rivalry in people with schizophrenia should investigate whether a similar trend (reduction of mean percept duration) is observable with an increase in people with schizophrenia of equivalent symptomatology. Our findings, however, should be viewed only as a first step toward controlling other variables, for example compliance, that may hinder the success of using rivalry rate as a reliable endophenotype for specific clinical populations. To have a practical impact our findings need to be replicated in clinical populations.

Overall, our data extended previous research showing that perceptual alternations relate to personality (Nagamine et al., 2007). The authors of that research, however, only focused on trait anxiety. Here, we used a broader approach, employing two personality taxonomies to demonstrate additional correlations between perceptual alternations and personality. Further research is required to determine the biological basis of the relationship found between personality and binocular rivalry.

A number of clinical studies have reported that a variety of psychiatric or neurological conditions (i.e., bipolar, schizophrenia, ADHD, autism, migraine) are associated with slower perceptual alternations. However, before now, it was unknown whether perceptual alternation rate correlates with other personality traits across individuals, in the general population.

Taking advantage of the individual differences approach utilized in this study, we were also able to determine that the proportion of mixed percept reported in the general population does not influence the relations found with personality. This finding is important because there are a lot of methodological differences in how different researchers treat (or entirely ignore) the mixed percept when they calculate rate.

In conclusion, we have shown that differences in rivalry dynamics correlate with other stable individual differences in personality. This result is important because it unveils new associated factors related to the striking degree of individual differences in rivalry alternation rate. Based on current models of binocular rivalry it is unclear what factors may underlie these links between individual differences in switch rate and personality traits. It will be interesting for future research to explore this further. From a practical point of view, if this data is replicated in a clinical population it would provide encouraging evidence that slower rivalry rates often seen in those groups are not a simple consequence of reduced conscientiousness/compliance and can be reliably used as a diagnostics tool.

## Ethics statement

Participants signed a consent form prior the testing phase and were able to withdraw from the project at any time. The study was approved by the University of Melbourne Human Research Ethics Committee, in accordance with the Declaration of Helsinki.

## Author contributions

All authors developed the study concept and contributed to the study design; AA collected the data; AA and LS performed the data analyses; AA drafted the manuscript; LS and OC provided critical revisions to the manuscript. All authors approved the final version of the manuscript for submission. All authors are accountable for all aspects of the work in ensuring that questions related to the accuracy or integrity of any part of the work are appropriately investigated and resolved.

### Conflict of interest statement

The authors declare that the research was conducted in the absence of any commercial or financial relationships that could be construed as a potential conflict of interest.
